# Organizational interventions and occupational burnout: a meta-analysis with focus on exhaustion

**DOI:** 10.1007/s00420-023-02009-z

**Published:** 2023-09-28

**Authors:** Isabelle Bes, Yara Shoman, Muaamar Al-Gobari, Valentin Rousson, Irina Guseva Canu

**Affiliations:** 1 Department of Occupational and Environmental Health, Center for Primary Cary and Public Health (Unisanté), University of Lausanne, Epalinges, Switzerland; 2 Quantitative Research Secteur, Unisanté, University of Lausanne, Lausanne, Switzerland

**Keywords:** Work-related stress, Exhaustion, Participatory intervention, Combined intervention, Controlled trial

## Abstract

**Purpose:**

To assess whether organizational interventions are effective to prevent or reduce exhaustion, the core dimension of occupational burnout.

**Methods:**

We searched in PubMed, EMBASE, PsycINFO, and Cochrane Library databases randomized and non-randomized controlled trials conducted among active workers and reporting the outcome as exhaustion score. We calculated the effect sizes using the pre-test–post-test control group design’s estimate. We used the random effects model in meta-analysis and Cochrane collaboration’s tool for interventions to assess the risk of bias. Overall quality of evidence was appraised using the GRADE.

**Results:**

From the 2425 identified records, we assessed 228 full texts for eligibility and included 11 original articles describing 13 studies, 11 on organizational interventions, and 2 on combined inventions. The interventions were participatory (*n* = 9), focused on workload (*n* = 2), or on work schedule (*n* = 2). The overall effect size was − 0.30 ((95% CI = − 0.42; − 0.18), I^2^ = 62.28%), corresponding to a small reduction in exhaustion with a very low quality of evidence. Combined interventions had a larger effect (− 0.54 (95% CI = − 0.76; − 0.32)) than organizational interventions. When split by type of intervention, both participatory interventions and interventions focused on workload had a benefic effect of exhaustion reduction, with an estimated effect size of − 0.34 (95% CI = − 0.47; − 0.20) and − 0.44 (95% CI = − 0.68, − 0.20), respectively.

**Conclusion:**

Interventions at combined level in workplaces could be helpful in preventing exhaustion. However, the evidence is still limited, due to a high heterogeneity between studies, bias potential, and small number of eligible studies. This calls for further research, using workload interventions at organizational level, especially in sectors with high risk of job stress and exhaustion.

**Supplementary Information:**

The online version contains supplementary material available at 10.1007/s00420-023-02009-z.

## Introduction

Occupational burnout is an occupation-related phenomenon recognized by the World Health Organization (WHO) in its international classification of diseases, but currently, burnout is not a diagnosis. Most of the conducted burnout research focused on burnout as a state and not a process (De Hert 2020), although, in the beginning, Freudenberger depicted the development of burnout in a model consisting of 12 stages (Freudenberger 1982). On the other hand, Maslach and colleagues presented burnout as a process of gradual fatigue, cynicism, and loss of commitment among social care professionals (Maslach and Leiter 1976). The etiology of burnout remains debated. Edu-Valsania et al. summarized six main theories of burnout development (Edu-Valsania et al. 2022). For example, according to the Organizational Theory, burnout is an outcome of organizational and work stressors associated with insufficient individual coping skills (Cox et al. 1993). Burnout starts with emotional exhaustion as a result of work stressors and then depersonalization develops as a coping technique against emotional exhaustion and leads to a low personal fulfillment (Cox et al. 1993). Therefore, interventions can prevent the burnout development by reducing exhaustion. Recently some organizations implemented different types of interventions in order to reduce employee’s exhaustion and prevent burnout development (Panagioti et al. 2017a).

Considering the heterogeneity in describing the etiology of burnout, it is not clear whether it should be considered a medical condition or not. Some countries recognize burnout as an occupational disease and have established more or less standardized, criteria, for its detection and management. This is notably the case in Sweden, Belgium and the Netherlands (Guseva Canu et al. 2019). Whether it is recognized as a disease or not, untreated burnout often leads to morbidity and is accompanied by depressive and anxiety symptoms (Koutsimani et al. 2019). In the most advanced stage, called severe or clinical, burnout is often confused with depression and presents a suicidal risk (van der Heijden et al. 2008). Some researchers have suggested that burnout could reflect a depressive state and not a distinctive entity (Bianchi et al. 2021). Burnout correlates with depression, specifically the exhaustion component of burnout; however, the most recent meta-analysis (Koutsimani et al. 2019) concluded that burnout and depression are two different constructs. An early detection of burnout would allow avoiding its health, economic and social consequences such as loss of productivity, absenteeism, high turnover and disengagement from active life (Aumayr-Pintar et al. 2018). The cost of physicians and veterinarians’ burnout in the United States is estimated between $1 and 2 billion annually (Han et al. 2019; Neill et al. 2022). To counter this phenomenon, organizations have attempted to prevent burnout through different types of interventions. Individual-level interventions aim help workers to cope with stress at the workplace using mainly behavioral and cognitive approaches or target individual knowledge (Dijxhoorn et al. 2021; Hepburn et al. 2021; Kuster et al. 2017). Organization-directed interventions are targeting structural changes such as schedule, workload or increasing job control and resources (Awa et al. 2010). Combined interventions, employ both individual and organization-level approaches and capture the benefits of targeting the workplace stressors while also improving employee resilience and coping strategies (Awa et al. 2010).

All existent burnout measures are multidimensional (Shoman et al. submitted; Shoman et al. 2021) and only few of them allow calculating an overall burnout score such as Copenhagen Burnout Inventory (CBI) (Kristensen et al. 2005) and Burnout Assessment Tool (BAT) (Schaufeli et al. 2020). While CBI only considers exhaustion, BAT and other burnout measures such as the Maslach Burnout Inventory (MBI) and Oldenburg Burnout Inventory (OLBI) consider additional dimensions (e.g., cynicism, depersonalization, or disengagement). Nevertheless, these dimensions are usually considered secondary as opposite to exhaustion. Exhaustion is the first symptom to develop and stands in a central position in burnout natural course (Maslach and Leiter 2016). From a prevention perspective, it is more effective to focus on the early symptoms rather than the more advanced ones (Kisling 2023). Moreover, exhaustion (both physical and emotional) is the only consensual characteristic of occupational burnout in workers according to the recent harmonized definition (Guseva Canu et al. 2021). Thus, by preventing exhaustion, one may prevent the worker from developing the other burnout symptoms such as cynicism or disengagement. As exhaustion dimension is measured by virtually all burnout scales, for the sake of homogeneity, we focused on it while measuring the effect size of interventions in the present meta-analysis. Indeed, the major drawback of the previously published meta-analyses is their very broad spectrum of studies and outcomes included resulting in a high between-study heterogeneity, result imprecision and inconclusiveness.

For instance, two meta-analyses (De Simone et al. 2021; Panagioti et al. 2017b) included both individual and organizational level interventions with mixed study designs and plethora of outcomes, making interpretation of the quality of evidence challenging. Conversely, two other meta-analyses (Thomas Craig et al. 2021; West et al. 2016) reduced the selection criteria to specific occupational groups in healthcare sector and are not generalizable beyond this sector. Finally, meta-analyses that limited the selection criteria to one burnout measurement scale (e.g., MBI (De Simone et al. 2021; Salvado et al. 2021)) are hardly generalizable to burnout constructs measured with other scales, especially those proven as more valid (Shoman et al. 2021). All the previously mentioned meta-analyses measured burnout as a multifaceted construct (including the exhaustion component). However, following the harmonized definition of burnout that states: “*In a worker, occupational burnout or occupational physical AND emotional exhaustion state is an exhaustion due to prolonged exposure to work-related problems*”, we decided to focus on the exhaustion component of burnout as discussed above. This definition is the consensual definition of occupational burnout as a clinical term, based on a systematic review, semantic analysis and expert consensus obtained using the Delphi method (Guseva Canu et al. 2021). It is the only definition in line with the existent definition of burnout in Systematized Nomenclature of Medicine—Clinical Term (SNOMED-CT), the only official controlled vocabulary of clinical terms and medical procedures (Dell'Oro et al. 2023).

Our objective was to evaluate the effectiveness of organizational interventions on occupational burnout prevention regardless the occupational sector aimed at identifying interventions with the strongest effect.

## Methods

### Protocol and registration

We conducted a systematic review and meta-analysis and adhered to the Preferred Reporting Items for Systematic Reviews and Meta-Analyses (PRISMA) guidelines for reporting the results (Page et al. 2021). The study protocol was registered on the international prospective register of systematic reviews (PROSPERO RecordID CRD42022357406).

### Literature searches

We designed a systematic search strategy and interrogated four databases: Medline (Pubmed), EMBASE, PsycINFO, and Cochrane Library for the period from journal inception and up to 12 September 2022. The full search strategy is available in details (Supplementary File S1). In PubMed, for instance, the search strategy comprised Medical Subject Heading Terms (MeSH) (e.g., “Burnout, Professional” [Mesh]), free text words (e.g., “emotional exhaust” [tiab]), Boolean terms (e.g., AND, OR) and truncations (e.g., work*) where necessary. In Embase, we used EMTREE terms, free text words, Boolean terms, proximity operators (e.g., Near/n), and truncations where necessary. Besides electronic searches, we manually searched reference lists of identified studies and prior systematic reviews and used the google search engine as well as the google scholar platform to identify additional eligible studies. There were no language or publication date restrictions.

### Eligibility of studies

Original articles conducted in adult workers of any occupation, with an intervention delivered at workplace either at organizational or combined levels to reduce or minimize workers’ exhaustion were eligible. We included original studies that reported the scores of exhaustion pre- and post-intervention. Some of these studies reported the scores of other dimensions of burnout but we did not consider these dimensions in this meta-analysis. To be included, studies should be randomized or non-randomized controlled trials (RCT or non-RCT). The latter were considered because often in occupational settings it is unethical and/or practically impossible to allocate the intervention randomly and blindly. In addition, to be included into the meta-analysis, the eligible studies must have measured burnout exhaustion dimension by a validated scale (Shoman et al. 2021) before and after the intervention. We excluded studies that were either testing an individual-oriented intervention, or without control group or had not reported burnout scale used and resulting scores.

All steps of the systematic review (from the double screening to the risk of bias assessment) were conducted by two reviewers independently (IB and MA). A third reviewer (IGC) was consulted in case of discrepancies which were discussed and solved consensually.

#### Literature screening, study inclusion and data abstraction

After excluding duplicates, the identified articles were first examined based on the title and abstract for eligibility assessment. Eligible and potentially eligible (uncertain) articles were then examined based on the full text. Studies fulfilling the inclusion criteria were included into the systematic review and data extraction. Key descriptive characteristics of the included studies were extracted, including the study design, characteristics of the study sample, occupational sector, sample size, age, gender, intervention type and content, alternative intervention (control), burnout measures/scales, outcomes measured, and follow-up duration.

#### Risk of bias assessment and evidence appraisal

We performed the risk of bias assessment using the Review Manager 5.3 Cochrane’s software risk of bias (Sterne et al. 2014). This tool allows the appraisal of the quality of included studies by rating seven domains: random sequence generation, allocation concealment, blinding of participants and personnel, blinding of outcome assessment, incomplete outcome data, selective reporting, and other bias. Each item in each domain was rated as low, unclear, or high risk of bias. Finally, each study was rated either as having an overall low, unclear, or high risk of bias, based on the overall judgment of the seven mentioned domains.

We assessed the overall quality of evidence following the Grading of Recommendations, Assessment, Development, and Evaluations (GRADE) approach (Guyatt et al. 2008). The GRADE consists of five domains: risk of bias, imprecision, inconsistency, indirectness, and publication bias. Following the GRADE, we started with the assumption that the level of evidence is high and downgraded it by one or two levels (e.g., from high to moderate) whenever necessary for each of the five domains of GRADE.

#### Missing data

We could not impute missing outcomes when they were not reported in the original studies. However, we contacted the corresponding authors of these studies to complete the extracted data and include the studies in the meta-analysis. In case we did not receive any answer from the authors, we had to exclude studies that did not report necessary data for the analysis. We calculated the standard deviations (SD) from the standard errors or confidence intervals, to estimate the SD differences.

#### Statistical analysis

We calculated the effect size for each study using Morris’s *d*_ppc2_ formula (Morris 2008):

$${\text{d}}_{{{\text{ppc2}}}} = {\text{cp }}\left( {\left( {{\text{Mpost}}.{\text{I}} - {\text{Mpre}}.{\text{I}}} \right) - \left( {{\text{Mpost}}.{\text{C}} - {\text{Mpre}}.{\text{C}}} \right)} \right)/{\text{SDpre}}$$

In his formula, cp is a correction factor equal to 1-(3/4(n I + n C -2)-1) where n I and n C are the numbers of participants in the intervention (I) and control (C) groups, respectively. The *d*_ppc2_ is a standardized difference of the differences of the pre- and post-intervention means in the intervention and control groups, divided by the pooled pre-intervention SD. Standard errors were calculated from the variance formula given in Supplementary File S2, enabling us to calculate 95% confidence intervals (95% CI) in each study. Of note, this variance formula involves a correlation rho between pre- and post-intervention measurements, which was not always reported. In some studies, we could retrieve rho from regression coefficients. When not available, we imputed the average value from the other studies (rho = 0.64).

The interpretation of the *d*_ppc2_ estimate is usually the same as of Cohen's *d* (Cohen 1988). However, this strictly numerical interpretation, separated from the context of the studies in a natural environment, is currently being questioned towards an interpretation in a discipline-specific manner (Cumming and Calin-Jageman 2016). Considering that organizational interventions are more in the realm of work and organizational psychology and use approaches that could be described as educational, *d*_ppc2_ cutoffs of 0.2, 0.5, and 0.8 are interpreted as small, medium, and large effects, respectively (Fritz et al. 2012).

We meta-analyzed the *d*_ppc2_ effect sizes and their 95% confidence intervals using a random effects model (DerSimonian and Laird 1986), which integrates an assumption that the different studies are estimating different, yet related, effects of the intervention. Heterogeneity among studies was measured via the *I*^2^ coefficient. We further performed subgroup analysis by the level of intervention (organizational vs combined), type of intervention (participatory, workload, and schedule), occupation of participants, the scale used in the study, and follow-up duration. We also performed sensitivity analysis by eliminating each study at a time from the analysis and assessing the effect by comparing the summary estimates (*d*_ppc2_) and the heterogeneity (*I*^2^) before and after the elimination of each study (Sutton et al. 2000).

Publication bias was assessed using both funnel plot and Egger regression (Egger et al. 1997) and Begg’s tests (Begg and Mazumdar 1994). We performed meta-regression to investigate how the above-mentioned study characteristics are related with the intervention effects.

All statistical analyses were performed using Stata statistical software, version 16 (StataCorp LP, Texas).

## Results

### Study selection

**Fig. 1:**
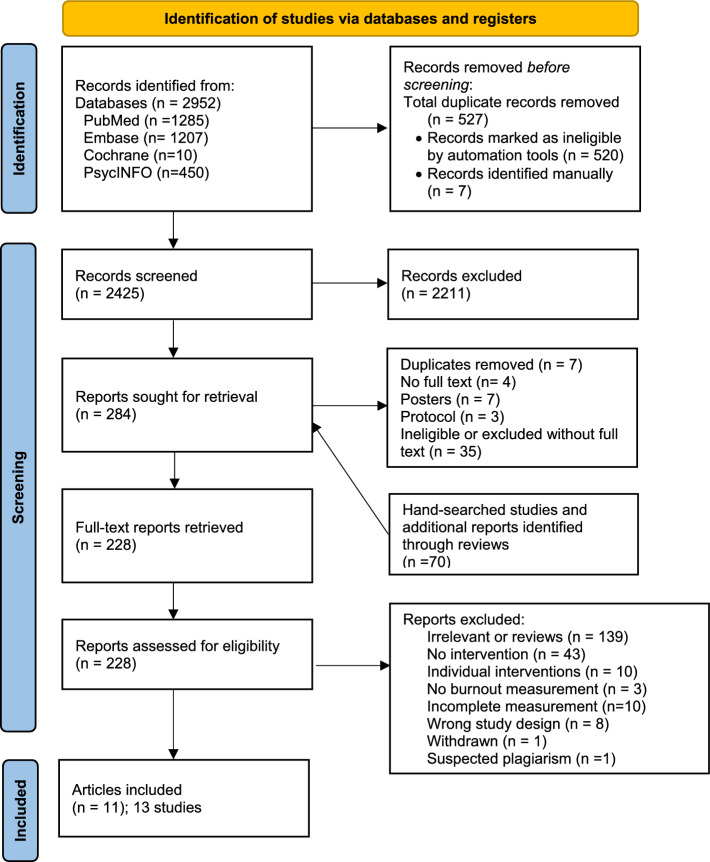
Flowchart of the included studies

We identified 2952 potentially relevant unique records from the literature search. We removed 527 duplicates and included 2425 records in the first screening based on titles and abstracts. In the second screening, we assessed 228 full-text records against inclusion criteria and excluded 215 studies for various reasons (e.g., reviews, individual interventions, wrong study design or no exhaustion estimate (Fig. 1)). The overall disagreement between the two reviewers was 16% and was solved by consensus between three reviewers. During the screening, we identified further 70 studies by hand search and from the screening of the reference list of the eligible articles.

Finally, we included 11 original articles, 2 of which involved 2 study samples (Gordon et al. 2018; Shea et al. 2014). Thus, we had 13 studies of 13 different samples which were meta-analyzed.

### Characteristics of included studies

The sample size of included studies ranged from 39 to 1173 workers with a median of 119. The participation of female workers ranged from 28 to 90% (Table 1). Twelve studies out of 13 included healthcare workers, such as nurses (Begg and Mazumdar 1994; Bourbonnais et al. 2011; Gordon et al. 2018; Melchior et al. 1996; van Weert et al. 2005), physicians (Adriaenssens et al. 2015; Gordon et al. 2018; Gregory et al. 2018; Shea et al. 2014) and other caregivers (Le Blanc et al. 2007; Leiter et al. 2011; Peterson et al. 2008). The remaining study was conducted on logistic workers (Demerouti et al. 2021). Burnout was measured at least twice, at baseline and at another measurement point after the end of intervention. Eight studies (62%) used the MBI in its original or translated version (Berg et al. 1994; Gregory et al. 2018; Leiter et al. 2011; Melchior et al. 1996; Shea et al. 2014; van Weert et al. 2005), four studies (31%) used OLBI (Demerouti et al. 2021; Gordon et al. 2018; Peterson et al. 2008) and one study used CBI (Bourbonnais et al. 2011), so we could extract the exhaustion score from all studies, to measure the outcome.

**Table 1:** Description of the included studies (*n* = 13)

Author, year	Design	Population	Sample size, mean age, % of women	Intervention (content and duration)		Intervention level	Intervention’s type	Exhaustion measure/scale	Exhaustion score baseline—control and intervention	Follow-up time points (months)	Intervention duration	Risk of bias
Berg (1994)	Non-RCT	Nurses	39;34;82	Implementation of a systematic clinical supervision plan for 12 months		Organizational	Participatory interventions	MBI	17 and 14	Baseline, 6 and 12 months	12 months	Unclear
Bourbonnais (2011)	Non-RCT	Nurses	613; NR; 82	An intervention team composed of members from 3 hospital units who identify problems and solutions in the framework of a participative intervention		Organizational	Participatory interventions	CBI	49.36 and 48.11	Baseline and 12 months	12 months	High
Demerouti (2021)	Non-RCT	Logistics employees	71;45; 43	The intervention consisted of a workshop to identify the self-chosen job crafting followed by 4 weeks of implementation and an evaluation session		Organizational	Participatory interventions	OLBI	2.03 and 2.21	Baseline and 2 months	4 weeks	Unclear
Gordon 1 (2018)	Non-RCT	Physicians	119; 51; 30	Impact of a specific job constructing intervention on health care professionals’ well-being and job performance		Organizational	Participatory interventions	OLBI	1.88 and 1.93	Baseline and 1.5 month	3 weeks	High
Gordon 2 (2018)	Non-RCT	Nurses	58; 36.2; 90	Impact of a specific job constructing intervention on health care professionals’ well-being and job performance		Organizational	Participatory interventions	OLBI	2.36 and 2.20	Baseline and 1.5 month	3 weeks	High
Gregory (2018)	Non-RCT	Physicians	69; NC; NC	Intervention’s focused on 4 clinics on workload by increasing human resources by 50%		Organizational	Workload	MBI	24.38 and 24.41	Baseline, 1, 2 and 3 months	6 months	Unclear
Le Blanc (2007)	Non-RCT	Healthcare workers	664; 36.2; 28	Take Care Intervention: a stress-management training; 6 monthly 3-h sessions where participants formed teams that collectively designed, implemented, evaluated, and reformulated plans of action to cope with most important stressors in their work situation		Organizational	Participatory interventions	MBI-HSS	1.46 and 1.54	Baseline, 6 and 12 months	6 months	Unclear
Leiter (2011)	Non-RCT	Healthcare workers	1173; 42.5; 86	Employees met with coworkers on their units on a weekly/biweekly basis to work on effective interpersonal interactions for 6 months		Organizational	Participatory Interventions	MBI-GS	2.73 and 3.21	Baseline and 6 months	6 months	High
Melchior (1996)	Non-RCT	Nurses	161; 34.8; 72	Nurses were assigned to patients as primary nurses with adequate feedback and support (advice on skills, support meeting between primary nurses and other health care specialists, training program and monthly evaluation)		Organizational	Participatory interventions	MBI	16.96 and 14.47	Baseline and 30 months	12 months	Unclear
Peterson (2008)	RCT	Healthcare workers	131; 51.7; 90	Reflecting peer-support group’s implementation: 2-h weekly working-group sessions, 10 times. All sessions started with a relaxation and was followed by a group meeting guided by an experienced group leader		Combined	Participatory interventions + individual	OLBI	3 and 3.03	Baseline and 12 months	2.5 months	Low
Shea 1 (2014)	RCT	Medical Interns	106; 27.5; 52	Effect of a 5-h period in which interns were expected to sleep during overnight call with rotation every 4 week in Philadelphia		Organizational	Schedule	MBI-HSS	25.48 and 23.91	Baseline and 1 month	11 months	Low
Shea 2 (2014)	RCT	Medical Interns	79; 28; 48.5	Effect of a 5-h period in which interns were expected to sleep during overnight call with rotation every 4 week in Pennsylvania		Organizational	Schedule	MBI-HSS	26.60 and 24.24	Baseline and 1 month	11 months	Low
Van Weert (2005)	Non-RCT	Nurses	128; 35; 59	Implementation of Snoezelen’s program for CNA: combined interventions to improve caregivers’ knowledge, skills		Combined	Workload + individual	MBI-NL	10.35 and 10.75	Baseline and 18 months	NC	Low
Overall level of evidence following the GRADE approach												
Studies assessed			Risk of bias		Imprecision	Inconsistency		Indirectness	Publication bias		Level of evidence	
All studies (13)			Yes		No	No		Yes	Yes		Very low	

^*^RCT randomized control trial, *non-RCT* non-randomized control trial, *MBI* Maslach Burnout Inventory, *CBI* Copenhagen Burnout Inventory, *OLBI* Oldenburg Burnout Inventory. *NR* not reported

Of the 13 included studies, 5 were classified to have an unclear risk of bias (Berg et al. 1994; Demerouti et al. 2021; Gregory et al. 2018; Le Blanc et al. 2007; Melchior et al. 1996), 4 had a high risk of bias (Bourbonnais et al. 2011; Gordon et al. 2018; Leiter et al. 2011), and 4 had a low risk of bias (Peterson et al. 2008; Shea et al. 2014; van Weert et al. 2005) (Table 1). The detailed results of the risk of bias assessment are presented in Supplementary Figure S1. Eleven studies were non-RCTs (Berg et al. 1994; Bourbonnais et al. 2011; Demerouti et al. 2021; Gordon et al. 2018; Gregory and Menser 2015; Le Blanc et al. 2007; Leiter et al. 2011; Melchior et al. 1996; van Weert et al. 2005) and 2 studies were RCTs (Peterson et al. 2008; Shea et al. 2014).

### Characteristics of interventions

Interventions varied considerably in their characteristics. Variability was found in many aspects, including intervention type, duration, follow-up duration, and outcome measure used. Interventions were classified either as organizational or as combined, with only two studies (Peterson et al. 2008; van Weert et al. 2005) in the latter category. We further classified the interventions according to their content, in interventions focused on work schedule (Shea et al. 2014), on workload (Gregory et al. 2018; van Weert et al. 2005) and participatory intervention (Berg et al. 1994; Bourbonnais et al. 2011; Demerouti et al. 2021; Gordon et al. 2018; Gregory et al. 2018; Le Blanc et al. 2007; Leiter et al. 2011; Melchior et al. 1996; Peterson et al. 2008) (Table 1). Shea et al. implemented an intervention to improve physicians’ schedule and sleep recovery (Shea et al. 2014). As the study was conducted in two different hospitals with distinct samples, we counted it as two studies. Two other studies were classified in the workload category (Gregory et al. 2018; van Weert et al. 2005). While Gregory and al. focused on increasing human resources to decrease workload (Gregory et al. 2018), Van Weert and al. implemented a program to reduce workload by improving certified nursing assistants’ knowledge about dementia (van Weert et al. 2005). Nine studies were classified as participatory interventions (Berg et al. 1994; Bourbonnais et al. 2011; Demerouti et al. 2021; Gordon et al. 2018; Le Blanc et al. 2007; Leiter et al. 2011; Melchior et al. 1996; Peterson et al. 2008). The duration of the interventions ranged from 3 weeks to 12 months with a mean of 6.5 months. One study did not specify its duration (van Weert et al. 2005). Follow-up duration ranged from 4 weeks to 3 years after the intervention (Table 1).

### Main meta-analysis results

**Fig. 2:**
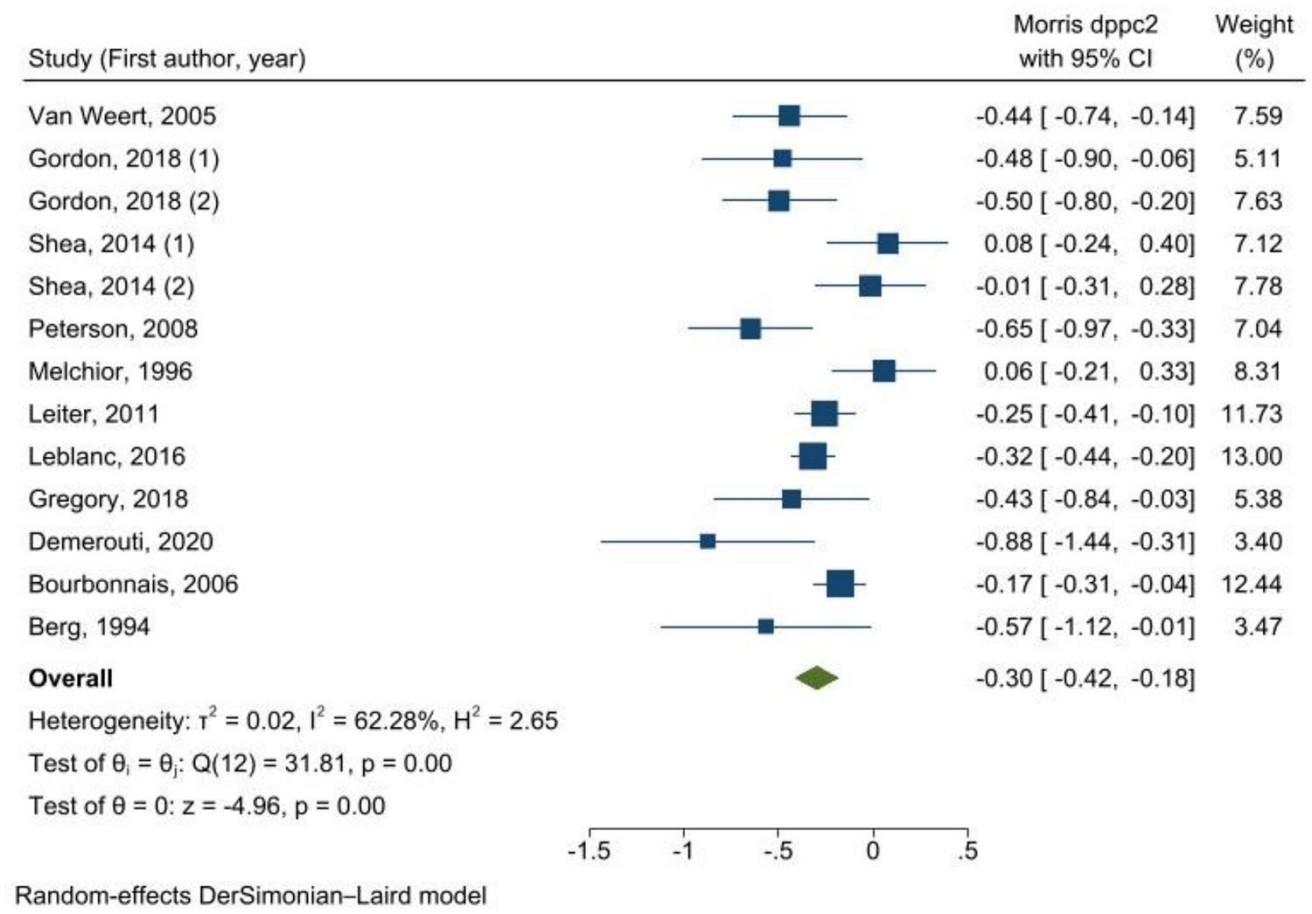
Meta-analysis of overall effect sizes from pre-test–post-test-control design (*d*_ppc2_) of organizational interventions to prevent exhaustion

An overall effect size was estimated at − 0.30 ((95% CI = − 0.42; − 0.18), *I*^2^ = 62.28%), corresponding to a moderate-weak effect on exhaustion reduction at the end of follow-up (Fig. 2). However, following the GRADE approach, the quality of evidence was very low.

#### Subgroup analyses

**Fig. 3:**
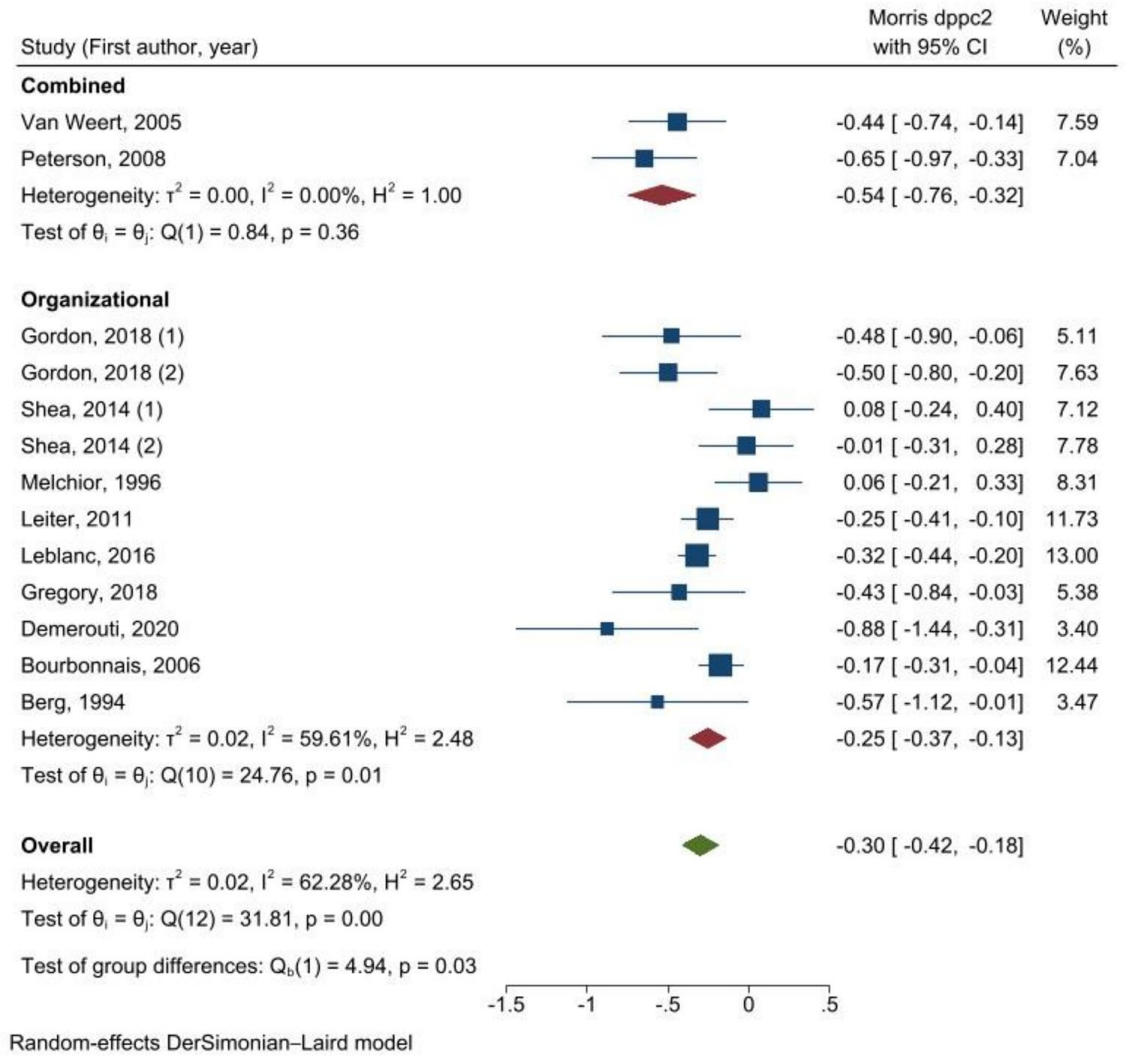
Meta-analysis of effect sizes from pre-test–post-test control design (*d*_ppc2_) of organizational interventions to prevent exhaustion, results of subgroup analysis by the level of intervention

**Fig. 4:**
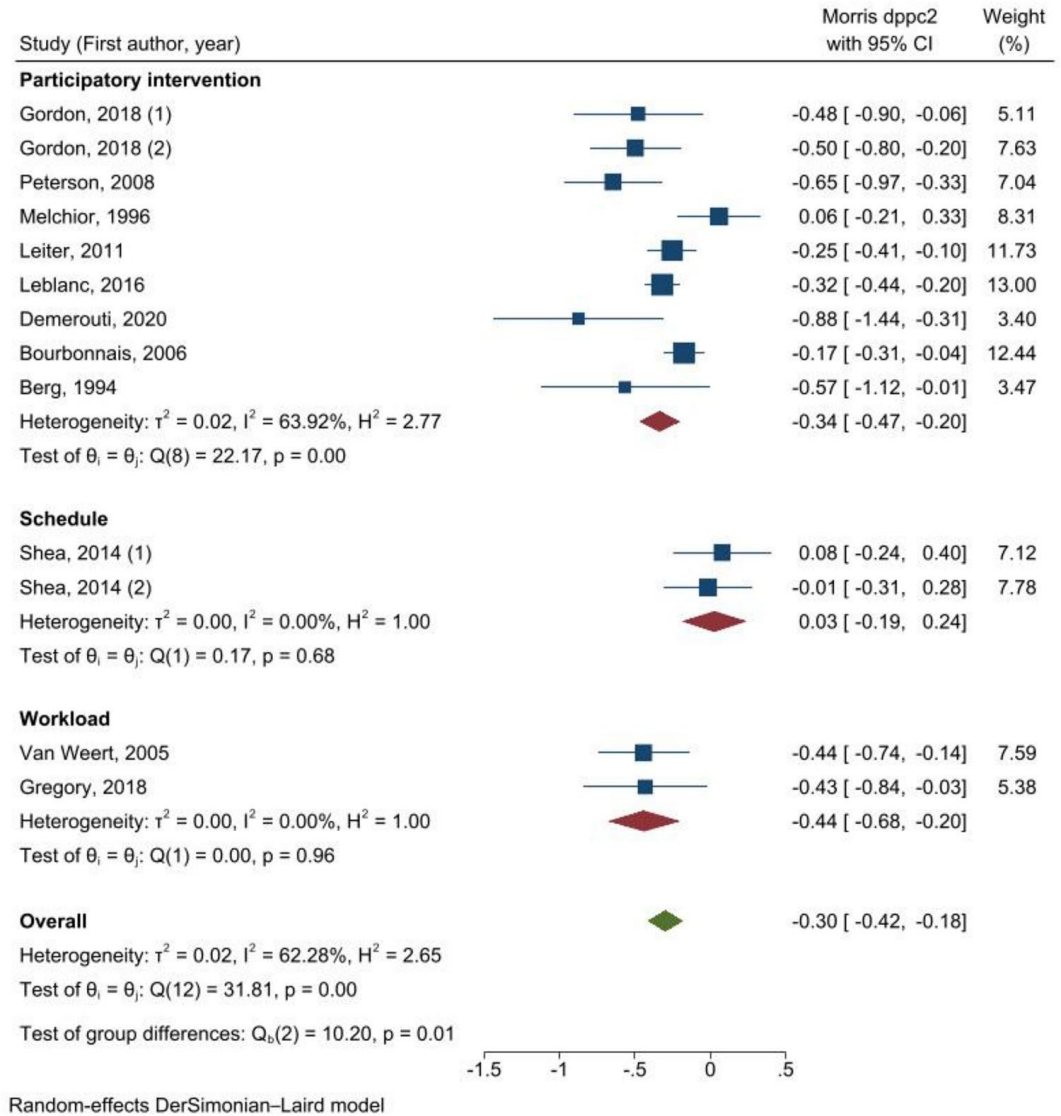
Meta-analysis of effect sizes from pre-test–post-test control design (*d*_ppc2_) of organizational interventions to prevent exhaustion, results of subgroup analysis by the type of intervention

Comparing the interventions by level (Fig. 3), we observed that the combined intervention has a large effect on reducing exhaustion score (*d*_ppc2_ = − 0.54 (95% CI = − 0.76; − 0.32)) based on 2 studies, while the organizational interventions have a rather small effect (*d*_ppc2_ = − 0.25 (95% CI = − 0.37; − 0.13), *I*^2^ = 59.61%) based on 11 studies. The subgroup analysis by the type of interventions showed statistically significant beneficial effects in all subgroups analyzed except the scheduling interventions (Fig. 4**)**. Interventions on workload showed a reduction in exhaustion score (*d*_ppc2_ = − 0.44 (95% CI = − 0.68; − 0.20)) based on two studies. This effect appears to be similar when combined with individual interventions (*d*_ppc2_ = − 0.44 (95% CI = − 0.74; − 0.14). The nine participatory interventions also reduced the exhaustion score overall (*d*_ppc2_ = − 0.34 (95% CI = − 0.47; − 0.20), *I*^2^ = 63.92%). Moreover, the effect size of participatory intervention increased when combined with an individual intervention (*d*_ppc2_ = − 0.65 (95% CI = − 0.97; − 0.33)).

In subgroup analysis based on follow-up duration, 1 month after the intervention, intervention impact was relatively small and only marginally significant. However, the analysis suggests a robust effect with a longer follow-up. Remarkably, the beneficial, though weak effect persisted after 12 months. (Supplementary Figure S2). Finally, the subgroup analysis by the scale used to measure exhaustion showed that interventions using the OLBI had a strong beneficial effect against exhaustion, whereas interventions using the MBI had a weak beneficial effect, less precise and more heterogeneous across studies (Supplementary Figure S3).

#### Sensitivity analysis, meta-regression, and publication bias

Based on a visual assessment of the funnel plot (Supplementary Figure S4) and the statistical tests results (Begg’s test *p*-value = 0.01, Egger’s test *p*-value = 0.19), we considered that potential for publication bias cannot be ruled out. The number of included studies being rather small, we kept meta-regression analysis univariate (Borenstein 2009). According to the results, some part of the heterogeneity can be explained by the level and type of intervention and participant’s occupation (Table 2). The results of sensitivity analyses changed neither the effect-size estimate, nor the heterogeneity.

**Table 2:** Results of univariate meta-regression analyses

Moderator	Beta coefficient	Standard error	P-value
Follow-up duration	0.01	0.01	0.32
Level of intervention	− 0.29	0.16	0.07
Type of intervention	0.14	0.08	0.07
Occupation (Healthcare vs non-healthcare)	− 0.60	0.33	0.07
Measurement scale	− 0.09	0.10	0.37

## Discussion

### Summary of main findings

This meta-analysis showed that organization-directed interventions and combined interventions were associated with moderate to small reductions in exhaustion score. Combined interventions were associated with higher reduction effects compared with organizational interventions. Interventions targeting workload were the most effective ones in reducing workers’ exhaustion, followed by participatory interventions. Interventions targeting work schedule had no effect on exhaustion and it seems unrelated to the risk of bias.

Examples of workload interventions are the one focusing on increasing the resources available to carry out the work and/or reducing the workload. Two studies replaced existing professionals dyads with more human resources (Gregory et al. 2018; Shaw et al. 2021); one study implemented remote access to more patients for 11 weeks (Romig et al. 2012) and another worked on improving employees’ knowledge to reduce their workload (van Weert et al. 2005). The success here seems to result from a better job resources balance. Participatory intervention’s category presented a high heterogeneity in its content. Employees are involved in five steps such as planning the intervention, identifying areas for improvement, developing action plans for improvement, implementing improvement initiatives, and evaluating the results in this type of interventions (Nielsen and Noblet 2018). This type of intervention recognizes that employees have the capacity to develop and implement their own solutions (Lavoie-Tremblay 2004). Employees’ sense of empowerment is strengthened, which in turn increases their sense of control and motivates them to engage in work (Quinn and Spreitzer 1997).

Interventions targeting workload could be effective in reducing exhaustion. One burnout development theory “i.e., Demands–Resources Theory” proposes that workers suffer from burnout if there is an imbalance between job demands and job resources (Bakker and Demerouti 2017). If the worker cannot recover from job demands (including but not limited to high workload), mental and physical exhaustion is prompted. Fatigue starts if demands exceed resources and if the imbalance between demands and resources is sustained over time, chronic fatigue occurs, followed by burnout. Hence, demands have a positive direct association with burnout, particularly exhaustion, while the presence of resources affects depersonalization by decreasing its use as a coping technique (Edu-Valsania et al. 2022).

### Limitations and strengths

The main limitations of this comprehensive meta-analysis were a relatively small number of included studies, a moderate between-study heterogeneity, and potential publication bias. The heterogeneity may be attributed to the clinical and methodological differences of the included studies, related to the choice of study population, intervention, burnout scale used, study design, and duration of intervention or follow-up. According to the sensitivity analysis, the observed heterogeneity cannot be attributed to a particular study, but to the differences observed in all the included studies (Aho 2020). Subgroup analyses reduced heterogeneity for some subgroups. However, the small number of included studies limited the scope of the subgroup analyses, where some subgroups included only in one or two studies.

The strong methodological framework that we followed in this meta-analysis is an important strength to mention. Other strengths are a precise outcome definition and quantification using Morris’s d_ppc2_. Indeed, exhaustion, retained as the main outcome in the meta-analysis, represents only one dimension of burnout but allowed us to harmonize the definition of the outcome. Most researchers agree that exhaustion is the core composing component of burnout (Schaufeli 2021). Exhaustion is cited in 12 of the 13 definitions that were included in the systematic review of burnout definitions (Guseva Canu et al. 2021). Although we recognize that exhaustion component of burnout can be emotional, physical, mental, or cognitive exhaustion (Schaufeli 2021), the original studies included in this meta-analysis did not differentiate these different types of exhaustion, which constitutes a limitation.

### Comparison with previous systematic reviews

Several meta-analyses were performed to examine the effectiveness of individual, combined and organizational interventions on physicians (Ahola et al. 2017; De Simone et al. 2021; Dreison et al. 2018; Panagioti et al. 2017b; Thomas Craig et al. 2021; West et al. 2016). Our results are in line with a recent meta-analysis (Thomas Craig et al. 2021) showing that combined interventions are more effective than organizational interventions to reduce burnout.

Considering the burnout/exhaustion score before intervention, as requested in Morris’s d_ppc2_ calculation, is also informative with respect to burnout/exhaustion severity. Moreover, it also comparing the studies according to their initial burnout level in study participants. For example, in four studies (Berg et al. 1994; Gregory et al. 2018; Shea et al. 2014), the measured scores corresponded to moderate exhaustion (with mean values of 17, 24, and 26, respectively) based on the MBI cutoffs (Maslach et al. 1997), while in two other studies (Melchior et al. 1996; van Weert et al. 2005), the scores corresponded to low exhaustion (14.30, 16.90, and 10.35, respectively). Among the studies that used the OLBI, two studies (Demerouti et al. 2021; Gordon et al. 2018) had low exhaustion scores. In contrast, a third study (Peterson et al. 2008) had a moderate score based on the authors’ threshold (Demerouti and Bakker 2008). To our knowledge, the burnout stage has been never considered in previous studies. Here, it might explain a part of between-study heterogeneity but can also present an interest for the intervention targeting and effect comparison between primary and secondary preventive intervention.

### Implications for researchers, clinicians, and policymakers

As the overall quality of evidence in this meta-analysis was graded as very low following the GRADE approach, despite moderate and large effects of some types of intervention, no practical recommendation can be suggested at this stage of knowledge. Conversely, several recommendations for future research can be formulated. As suggested previously (Thomas Craig et al. 2021), the studies that combined individual and organizational interventions lead to a larger effect in reducing exhaustion domain in occupational burnout. To confirm this, future study should consider having three arms or groups, with an intervention on organizational level along, the same intervention coupled with one on individual level and no intervention (control group). Measuring the outcome (at least the exhaustion score) at baseline and provide its clinical interpretation in terms of severity) should be systematically done in all groups and all future studies to make them more informative with respect to the stage of burnout and type of intervention (i.e., primary versus secondary). A more systematic and harmonized definition of the time points for the effect assessment is recommended to reduce the heterogeneity when comparing the different interventions. It will also inform the duration of the intervention’s effect and if necessary, a buster planning. Primary interventions aim to preserve the employee's resources and avoid burnout, while secondary intervention aims to prevent worsening of burnout symptoms in employees already affected by burnout at a low or moderate stage (LaMontagne et al. 2007). Finally, it worth to remind that the prior registration of the interventional study protocol and result publication regardless the trial conclusion should be compulsory to prevent publication and selection bias resulting in a lacunary evidence assessment. We are aware that publication of non-RCTs is challenging since they are supposed to be more prone to bias compared to RCTs, however, when considering the occupational filed, we need to circumvent this obstacle and promote methodologically strong and sound research even if the study design is not an RCT. For this, guidelines and methods appropriate to quantitatively assess the direction and magnitude of bias due to the non-RCT design particularities (e.g., impossibility of allocation randomization and concealment, spillover effect) are necessary, in a similar way they were developed for observational studies (Schubauer-Berigan et al. 2023).

## Conclusion

Interventions at combined level in workplaces could be helpful in preventing exhaustion, the first and core component of occupational burnout. However, the evidence is still very low, due to a moderate heterogeneity between the studies, bias potential, and moderate to small effect size. This calls for further studies and methodological efforts to make the non-RCTs valid and reliable, given their relevance to inform and improve occupational health research.

### Supplementary Information

Below is the link to the electronic supplementary material.

Supplementary file1 (DOCX 335 KB)

## Data Availability

All data are available in the manuscript and its supported files. Any more information can be requested from the corresponding author.
