# Supplementary material for: Organizational interventions and occupational burnout: a meta-analysis with focus on exhaustion
Source: Int Arch Occup Environ Health. 2023 Sep 28;96(9):1211–23. doi: 10.1007/s00420-023-02009-z (PMC10560169; doi:10.1007/s00420-023-02009-z)
Supplement: Supplementary file 1 — Supplementary file1 (DOCX 335 KB) [file 420_2023_2009_MOESM1_ESM.docx]

**Supplementary Files S1. The search string in three databases: PubMed. EMBASE, and PsycINFO**

***The search string in PubMed***
 ("Burnout, Professional"[Mesh] OR "professional exhaustion"[tiab] OR ("Burnout, Psychological"[Mesh:NoExp] OR burnout[tiab] OR "burn-out"[tiab] OR "emotional exhaust*"[tiab] OR "emotionally exhaust*"[tiab]) AND (job[tiab] OR jobs[tiab] OR occupation*[tiab] OR profession*[tiab] OR staff*[tiab] OR work*[tiab] OR employ*[tiab] OR colleague*[tiab])) AND ("Organizational Innovation"[Mesh] OR "Organization and Administration"[Mesh:NoExp] OR organisation* [tiab] OR organization* [tiab]) AND (intervention* [tiab] OR prevent*[tiab])

***The search string in EMBASE***

('professional burnout'/de OR 'professional exhaustion':ti,ab
OR 'burnout'/de OR burnout:ti,ab OR 'burn-out':ti,ab OR (emotion* NEAR/2 exhaust*):ti,ab)

AND

(job*:ti,ab OR occupation*:ti,ab OR profession*:ti,ab OR staff*:ti,ab OR work*:ti,ab OR employ*:ti,ab OR colleague*:ti,ab)

AND

organi?ation*:ti,ab

AND

(prevention*:ti,ab OR intervent*:ti,ab)

***The search string in PsycINFO***
((MeSH: burnout OR burnout OR burn-out OR (emotion* adj2 exhaust*)) AND (job* OR occupation* OR profession* OR staff* OR work* OR employ* OR colleagu*) AND (organisation OR organization) AND (intervent* OR prevent*))

AND Publication Type: Peer Reviewed Journal

***Keywords used for Cochrane database:***  Burnout; professional burnout

**Supplementary Files S2. Formula to compute the varaince of the effect size (d_ppc2_)**


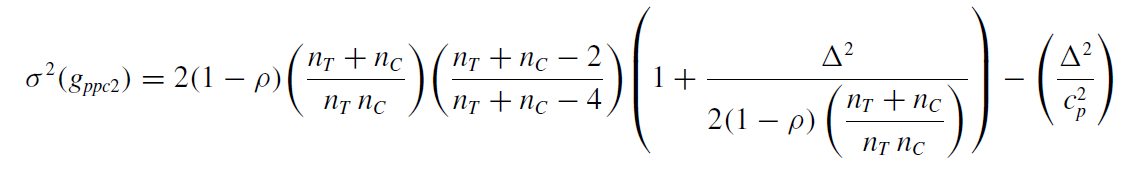


*Note: P: correlation coefficient,* n T: number of participants in the treatment (here intervention) group*,* n C: number of participants in the control group*, cp: correction factor corresponding to the formula: 1-(3/4(n T +n.C -2)-1), and Δ: the difference between the standardized mean change for the treatment and control groups*


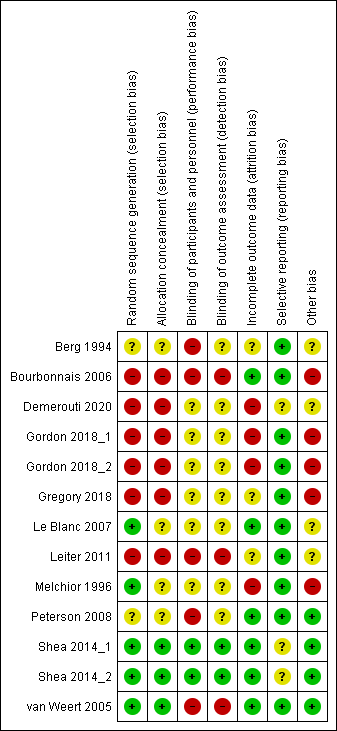


**Supplementary Figure S1. Results of risk of bias assessment**


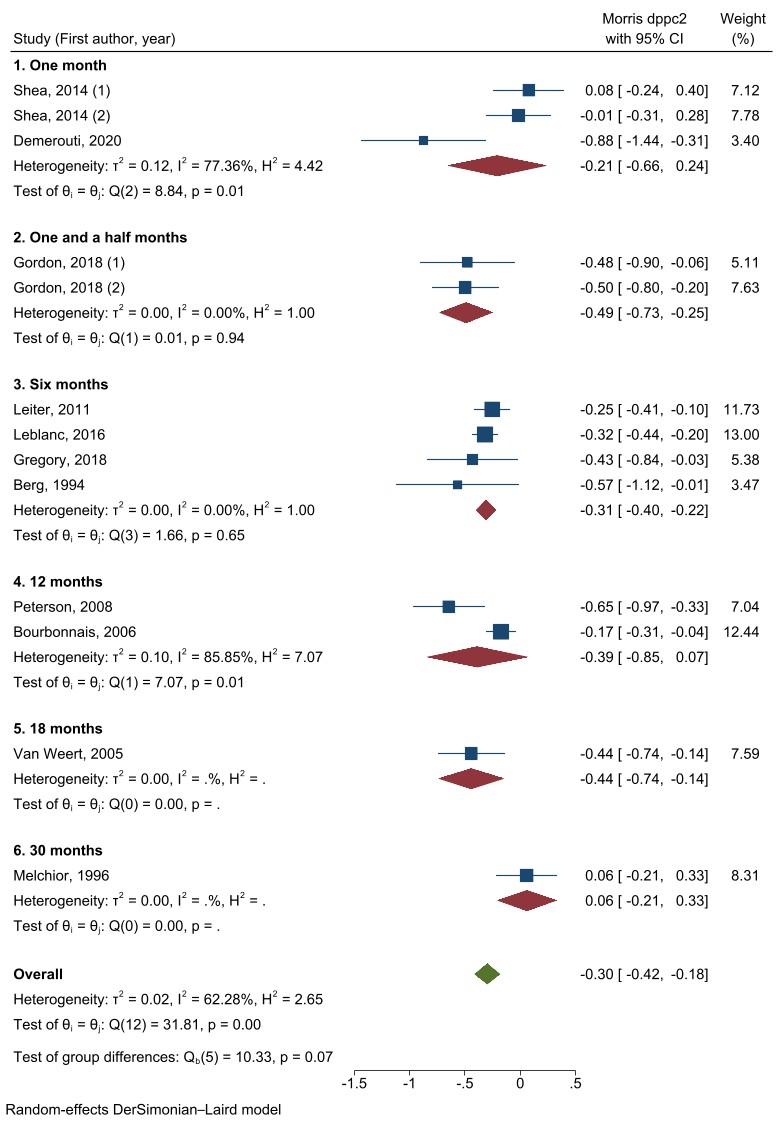


**Supplementary Figure S2. Meta-analysis of effect sizes from pretest-posttest-control design (dppc2) of organizational directed interventions to reduce or prevent occupational burnout, results of subgroup analysis by the follow-up duration**


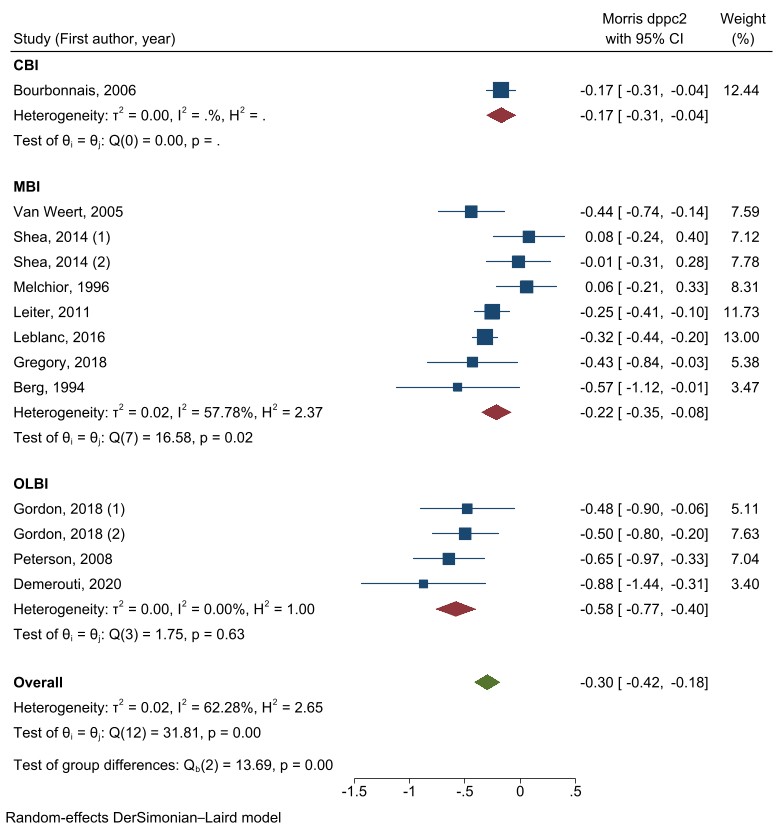


**Supplementary Figure S3. Meta-analysis of effect sizes from pre-test - post-test control design (d_ppc2_) of organizational directed interventions to reduce or prevent occupational burnout, results of subgroup analysis by the scale used to measure burnout**


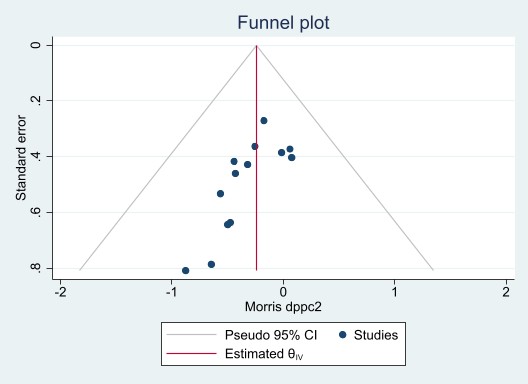


**Supplementary Figure S4. Funnel plot**
